# Management of potentially life-threatening emergencies at 74 primary level hospitals in Mongolia: results of a prospective, observational multicenter study

**DOI:** 10.1186/s12873-017-0127-4

**Published:** 2017-05-08

**Authors:** Naranpurev Mendsaikhan, Davaa Gombo, Ganbold Lundeg, Christian Schmittinger, Martin W. Dünser

**Affiliations:** 1 Intensive Care and Anesthesiology Department, Intermed Hospital, Chinggis Avenue 41, Duureg 15, Ulaanbaatar, 17040 Mongolia; 2 Public Health School, Mongolian National University of Medical Sciences, Ulaanbaatar, Mongolia; 3 Division of Emergency Medicine and Anesthesia, Mongolian National University of Medical Sciences, Ulaanbaatar, Mongolia; 4 Department of Anesthesiology, Kantonspital Luzern, Luzern, Switzerland; 5 Department of Critical Care, University College of London Hospital, London, NW1 2BU UK

**Keywords:** Emergency medicine, Primary level hospital, Hospital capacities, Mongolia

## Abstract

**Background:**

While the capacities to care for and epidemiology of emergency and critically ill patients have been reported for secondary and tertiary level hospitals in Mongolia, no data exist for Mongolian primary level hospitals.

**Methods:**

In this prospective, observational multicenter study, 74 primary level hospitals of Mongolia were included. We determined the capacities of these hospitals to manage medical emergencies. Furthermore, characteristics of patients presenting with potentially life-threatening emergencies to these hospitals were evaluated during a 6 month period.

**Results:**

An emergency/resuscitation room was available in 62.2% of hospitals. One third of the study hospitals had an operation theatre (32.4%). No hospital ran an intensive care unit or had trained emergency/critical care physicians or nurses available. Diagnostic resources were inconsistently available (sonography, 59.5%; echocardiography, 0%). Basic emergency procedures (wound care, 97.3%; foreign body removal, 86.5%; oxygen application, 85.2%) were commonly but advanced procedures (advanced cardiac life support, 10.8%; airway management, 13.5%; mechanical ventilation, 0%; renal replacement therapy, 0%) rarely available. During 6 months, 14,545 patients were hospitalized in the 74 study hospitals, of which 8.7% [*n* = 1267; median age, 34 (IQR 18–53) years; male gender, 54.4%] were included in the study. Trauma (excl. brain trauma) (20.4%), acute abdomen (16.9%) and heart failure (9.6%) were the most common conditions. Five-hundred-thirty patients (41.8%) were transferred to a secondary level hospital. The hospital mortality of patients not transferred was 3.2%.

**Conclusions:**

Capacities of Mongolian primary level hospitals to manage life-threatening emergencies are highly limited. Trauma, surgical and medical conditions make up the most common emergencies. In view of the fact that almost half of the patients with a potentially life-threatening emergency were transferred to secondary level hospitals and the mortality of those hospitalized in primary level hospitals was 3.2%, room for improvement is clearly evident. Based on our findings, improvements could be obtained by strengthening inter-hospital transfer systems, training staff in emergency/critical care skills and by making mechanical ventilation and advanced life support techniques available at the emergency rooms of primary level hospitals.

**Electronic supplementary material:**

The online version of this article (doi:10.1186/s12873-017-0127-4) contains supplementary material, which is available to authorized users.

## Background

Mongolia is a land-locked lower-middle-income country in Central Asia. It is home to approximately three million people with a life expectancy of 69.3 years at birth [1]. Emergency and critical care medicine is a young medical specialty in the country. The care of acutely and critically ill patients faces serious limitations in human and material resources [2, 3]. The epidemiology of critical illness in Mongolian intensive care units is comparable to Western countries with a high prevalence of stroke, liver failure and traumatic brain injury [4]. Mortality of a general intensive care population was substantial ranging around 25% [4, 5]. Health care facilities in Mongolia are separated into three different levels of care. Intensive care services are only available in secondary and tertiary level hospitals [6]. So far, however, no data on the capacity of primary level hospitals in Mongolia to manage patients with life-threatening emergencies have been published. In addition, the epidemiology of patients with medical emergencies presenting to these hospitals has not been defined.

In this study we sought to determine the capacities of a cohort of 74 out of 274 Mongolian primary level hospitals to manage medical emergencies. We also evaluated characteristics of patients presenting to these hospitals with potentially life-threatening emergencies during a 6 month time period.

## Methods

**Fig. 1:**
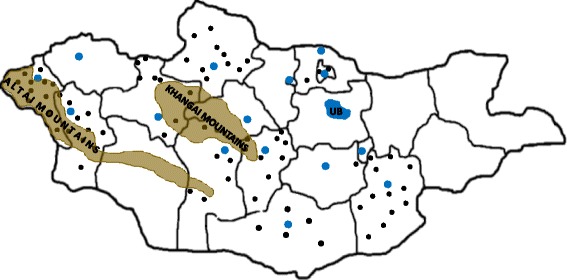
Map of Mongolia indicating the localization of the 74 primary level hospitals participating in this study (*black dots*), secondary level referral hospitals (*blue dots*), the capital city of Ulaanbaatar (*blue blot*), and major geographical barriers (*light brown*)

This analysis was designed as a multicenter prospective observational cohort study. It was conducted in 74 randomly selected primary level hospitals located in eleven of twenty-one Mongolian provinces (Fig. 1). The study protocol was reviewed and approved by the Ethics Committee of the Mongolian National University of Medical Sciences. Given that only anonymized data were collected and no study-related changes to patient management were performed, written informed consent was waived.

### Evaluation of study hospitals’ capacities to manage medical emergencies

During site visits before patient recruitment started, the study hospitals’ capacity to manage medical emergencies was evaluated by discussion with the physician-in-charge and confirmed by on-site inspection by one of the study team. We chose to determine the capacity to manage selected medical emergencies primarily based on the availability of trained staff, hospital structures and equipment, as the ability of individual physicians to perform such emergency procedure was difficult and unreliable to test. The following data were collected using a standardized report form (Additional file 1): number of hospital beds, availability of trained staff (emergency or critical care physicians and nurses), hospital structures (emergency/resuscitation room, delivery room, operating theatre, intensive care unit), selected diagnostic resources (sonography and echocardiography machine, laboratory facilities), and possibility to perform specific emergency procedures (wound care, burn wound care, foreign body removal (all foreign bodies except those in deep tissues/organs or the gastrointestinal tract), abscess drainage, fracture management, advanced cardiac life support, oxygen administration, airway management, cardioversion, thoracocentesis, paracentesis, pericardiocentesis, blood transfusion, non-invasive or invasive mechanical ventilation, central venous catheter insertion, renal replacement therapy).

### Characteristics of patients presenting with potentially life-threatening emergencies

During 6 months (March 1 until August 31, 2015), the following data were collected from all patients presenting with a potentially life-threatening emergency to one of the study centers: age, gender, admission diagnosis, the Modified Early Warning Score [7], the South African Trauma Score (in case of trauma) [8], need for emergency surgery, need for transfer to a secondary level hospital, length of stay in the study hospital, and hospital mortality. Admission diagnoses were retrospectively grouped into seven diagnostic categories. The reason for not transferring patients to a secondary level hospital was collected for all patients hospitalized in one of the study hospitals. In case of death at the primary level hospital, the suspected cause of death was documented.

Potentially life-threatening emergencies were defined as by the clinical judgement of the attending physician. In general, these patients required either hospitalization in one of the study hospitals or transfer to a secondary level hospital for emergency specialist care. Patients who presented to one of the study hospitals with a non-emergency condition or an emergency condition that was not serious enough to require hospitalization were not included.

### Statistical Analysis

The SPSS software package was used for all statistical analyses (SPSS Inc; SPSS IBM, Chicago, Illinois, United States of America). Descriptive methods were used to present data with categorical variables given as absolute numbers with percentage and continuous variables as median values with interquartile ranges. Comparisons between patients hospitalized in one of the study hospitals and patients transferred to secondary level hospitals as well as between different age groups were made using the *Chi* ^*2*^-test, Mann-Whitney-*U* test, Fisher’s Exact test or an analysis of variance, as appropriate. *P*-values <0.05 were considered to indicate statistical significance.

## Results

Capacities of the study hospitals to manage medical emergencies are summarized in Table 1. Restrictions were detected at multiple levels including a widespread lack of hospital structures such as emergency or resuscitation rooms, the unavailability of medical staff specifically trained in the management of life-threatening conditions, as well as the inconsistent availability of diagnostic resources. While basic emergency medical procedures such as wound care, foreign body removal and oxygen administration were offered by the majority of study hospitals, only one in ten hospitals could provide advanced cardiac life support, airway management or blood transfusion. Not a single study hospital reported to have the possibility to mechanically ventilate acutely or critically ill patients.

**Table 1:** Study hospitals’ capacities to manage medical emergencies

*n*	74
Number of beds (n)	10 (5–50)
Availability of hospital structures (n/%)	
*Emergency*/*resuscitation room*	46 (62.2)
*Delivery room*	72 (97.3)
*Operation room*	24 (32.4)
*High dependency unit*/*intensive care unit*	0
Availability of trained staff (n/%)	
*Emergency or critical care physician*	0
*Emergency or critical care nurse*	0
Availability of selected diagnostic resources (n/%)	
*Sonography*	44 (59.5)
*Echocardiography*	0
*Possibility to measure full blood count*	46 (62.2)
*Glucometer*	69 (93.2)
*Possibility to perform a urine analysis*	41 (55.5)
*Blood gas analyzer*	0
Possibility to perform selected emergency procedures (n/%)	
*Wound care*	72 (97.3)
*Burn wound care*	71 (95.9)
*Foreign body removal*	64 (86.5)
*Abscess drainage*	48 (64.9)
*Fracture management*	57 (77)
*Advanced cardiac life support*	8 (10.8)
*Oxygen application*	63 (85.2)
*Airway management*	10 (13.5)
*Cardioversion*	1 (1.4)
*Thoracocentesis*	6 (8.1)
*Paracentesis*	12 (16.2)
*Pericardiocentesis*	0
*Blood transfusion*	8 (10.8)
*Non*-*invasive mechanical ventilation*	0
*Invasive mechanical ventilation*	0
*Central venous catheter insertion*	2 (2.7)
*Renal replacement therapy*	0

Data are given as median values with range (minimum-maximum), if not otherwise indicated

During the observation period, 14,545 patients were hospitalized in the 74 study hospitals, of which 1267 patients (8.7%) were considered to suffer from a potentially life-threatening emergency and were enrolled into the study (Table 2). Table 3 presents the fifteen most common medical conditions of these patients. All patients were either hospitalized in one of the study hospitals (*n* = 737, 58.2%) or transferred to a secondary level hospital (*n* = 530, 41.8%). Patients transferred to secondary level hospitals were more frequently male, younger, differed in the diagnostic categories, had a higher Modified Early Warning Score and South African Trauma Score, and required emergency surgery more often than patients hospitalized in one of the study hospitals (Table 4). Table 5 presents clinical characteristics of patients stratified into different age groups.

**Table 2:** Demographic and clinical data of the study population

*n*	1267
Age (years)	34 (18–53)
Male gender (n/%)	690 (54.4)
Diagnostic categories (n/%)	
*Trauma*	325 (25.7)
*Surgical* (*non*-*trauma*)	245 (19.3)
*Medical*	226 (17.8)
*Neurological*	120 (9.5)
*Infection*	80 (6.3)
*Obstetrical*	17 (1.3)
*Other*	254 (20)
Modified Early Warning Score (pts)	3 (2–5)
South African Trauma Score (pts)	1 (1–2)
Need for emergency surgery (n/%)	132 (10.4)
Transfer to level II hospital (n/%)	530 (41.8)
Reason for not transferring (n/%)	
*Condition manageable at primary level hospital*	657 (89.1)
*Physician at secondary level hospital discouraged transfer*	26 (3.5)
*Patient too unstable*	23 (3.1)
*Transport not possible*	21 (2.8)
*Family disagreed*	10 (1.4)
Length of hospital stay (days)	5 (1–8)
Hospital mortality (n/%)	41 (3.2)
Cause of death (n/%)	
*Respiratory failure*	12 (29.3)
*Shock*	11 (26.8)
*Coma*	8 (19.5)
*Multiple organ dysfunction*	8 (19.5)
*Not given*	2 (4.9)

Data are given as median values with interquartile ranges, if not otherwise indicated

**Table 3:** Description of the 15 most common emergency conditions

Admission diagnosis (n/%)	All patients
*n*	1267
Trauma (excl. brain trauma)	258 (20.4)
Acute abdomen	214 (16.9)
Acute or acute-on-chronic heart failure	122 (9.6)
Brain trauma	67 (5.3)
Stroke (ischemic or hemorrhagic)	66 (5.2)
Community-acquired pneumonia	64 (5.1)
Metabolic coma	54 (4.3)
Cancer-related condition	44 (3.5)
Respiratory failure	35 (2.8)
Acute or acute-on-chronic kidney failure	31 (2.4)
Surgery-related condition	22 (1.7)
Pre-eclampsia/eclampsia	16 (1.3)
Decompensated liver cirrhosis	14 (1.1)
Infection (excl. community-acquired pneumonia and tuberculosis)	14 (1.1)
Miscellaneous	246 (19.4)

**Table 4:** Differences in characteristics of patients hospitalized in study centers and patients transferred to secondary level hospitals

	Patients hospitalized	Patients transferred	*p*-value
*n*	737	530	
Age (years)	38 (20–55)	32 (17–48)	0.005^a^
Male gender (n/%)	365 (49.5)	325 (61.3)	<0.001^a^
Diagnostic categories (n/%)			
*Trauma*	146 (19.8)	179 (33.8)	<0.001^a^
*Surgical* (*non*-*trauma*)	101 (13.7)	144 (27.2)	<0.001^a^
*Medical*	173 (23.5)	53 (10)	<0.001^a^
*Neurological*	69 (9.4)	51 (9.6)	0.7
*Infection*	51 (6.9)	29 (5.5)	0.41
*Obstetrical*	7 (0.9)	10 (1.9)	0.13
*Other*	190 (25.8)	64 (12.1)	<0.001^a^
Modified Early Warning Score (pts)	3 (2–5)	3 (2–5)	0.04^a^
South African Trauma Score (pts)	2 (1–2)	1 (1–2)	0.02^a^
Need for emergency surgery (n/%)	54 (7.3)	78 (14.7)	<0.001^a^
Length of hospital stay (days)	5 (1–8)	data not available	
Hospital mortality (n/%)	41 (3.2)	data not available	

Data are given as median values with interquartile range, if not otherwise indicated

^a^significant difference between age groups

**Table 5:** Differences in study patient characteristics between age groups

	<28 days	1–5 years	5–18 years	>18 years
*N* (%)	36 (2.8)	86 (6.8)	174 (13.7)	971 (76.6)
Male gender (n/%)	22 (61.1)	43 (50)	115 (66.1)	510 (52.5)
Diagnostic categories (n/%)				
*Trauma*	0	23 (26.7)	70 (40.2)	232 (23.9)
*Surgical* (*non*-*trauma*)	4 (11.1)	16 (18.6)	54 (31)	171 (17.6)
*Medical*	1 (2.8)	2 (2.3)	8 (4.6)	215 (22.1)
*Neurological*	8 (22.2)	4 (4.7)	12 (6.9)	96 (9.9)
*Infection*	14 (38.9)	22 (25.6)	12 (6.9)	32 (3.3)
*obstetrical*	0	0	0	17 (1.8)
*other*	9 (25)	19 (22.1)	18 (10.3)	208 (21.4)
Transfer to level II hospital (n/%)	14 (38.9)	27 (31.4)	94 (54)	395 (40.7)
Length of hospital stay (days)	7 (1–10)	7 (1–7)	7 (4.25–8)	7 (1–10)
Hospital mortality (n/%)	1 (2.8)	3 (3.5)	0	37 (3.8)
Cause of death (n/%)				
*Respiratory failure*	1 (100)	1 (33.3)	0	10 (27)
*Shock*	0	2 (66.7)	0	9 (24.3)
*coma*	0	0	0	8 (21.6)
*Multiple organ dysfunction*	0	0	0	8 (21.6)
*Not given*	0	0	0	2 (5.4)

Data are given as median values with interquartile ranges, if not otherwise indicated

## Discussion

One key finding of this study is that the capacities of 74 Mongolian primary level hospitals to manage life-threatening emergencies are highly limited. As information on the availability of capacities was collected from the physician-in-charge of each study site, it appears unlikely that our results have relevantly under-estimated the true capacities of the participating study hospitals to manage emergency patients. Nonetheless, documentation of available resources and capacities during the management of individual emergency cases may have provided more robust results.

Our study focused on the management of patients with potentially life-threatening emergencies. Given that only 8.7% of all patients hospitalized in the study hospitals or approximately ~3 patients per month per study center were included, our study cannot provide information on the management of non-life-threatening emergencies. The patient population included was young compared to reports from emergency departments in high-income countries [9], but comparable to studies from other low- and middle-income countries [10, 11]. Affecting one quarter of patients, particularly children, trauma was the most common diagnosis followed by surgical and medical conditions. Interestingly, emergencies due to infectious or obstetrical pathologies were uncommon. A relevant portion of emergencies due to infection was, however, seen in neonates and children under the age of 5 years. Since physicians at each study site determined whether the condition of a patient was considered a potentially life-threatening emergency or not, it is conceivable that certain diseases (e.g. infections in adults) may be under-represented in our study.

The in-hospital mortality of emergency patients who were not transferred to a secondary level hospital but cared for in one of the study hospitals was 3.2%. This number must be interpreted in light of the fact that almost half of all study patients were transferred to a higher level hospital. The disease course of these patients was not followed-up in our study making comparisons with mortality data reported by other authors difficult [9–13]. As shown in Table 4, physicians in primary level hospitals were more prone to transfer younger and male patients who suffered from either trauma or a surgical disease and had a higher disease severity. In about 10% of study patients transfer to a secondary level hospital was attempted but not performed because the transport was not possible or the patient considered too unstable. The results of our study support the need to strengthen inter-hospital transport systems in Mongolia. However, long and seasonally difficult to overcome distances between primary and secondary level hospitals in Mongolia, one of the least densely populated countries in the world, dictate that primary level hospitals should also be able to stabilize patients with life-threatening emergencies. Our results suggest key interventions to improve the management of patients with life-threatening emergencies at these hospitals. As it is unrealistic to staff primary level hospitals with emergency or critical care specialists, training of physician and nursing staff in basic emergency care appears pragmatic. Telemedicine has proven beneficial in critical care medicine in high-income countries [14] and deserves testing in low- and lower-middle income countries such as Mongolia, too. The fact that respiratory failure, shock and coma were the most common causes of death in this study population implies that provision of oxygen, (non-invasive) mechanical ventilators and advanced cardiac life support techniques could reduce morbidity and mortality of patients with life-threatening emergencies. Irrespective of the techniques implemented, adequate staff training appears essential. Systematic and focused courses on emergency and critical care medicine for physicians and nurses have been conducted successfully in resource-limited settings [15, 16].

Our study has additional limitations. First, we collected data from only 74 of all 274 primary level hospitals in Mongolia. Although it may be assumed that capacities and patient characteristics are similar in primary level hospitals which were not included in this survey, our study results cannot be extrapolated to all primary level hospitals in Mongolia. Second, we enrolled patients during 6 months spanning the time period from March until August. Seasonal variations of selected emergency conditions (e.g. trauma in summer, infection in winter) may have remained undetected by our analysis.

## Conclusions

Capacities of Mongolian primary level hospitals to manage life-threatening emergencies are highly limited. Trauma, surgical and medical conditions make up the most common emergencies. In view of the fact that almost half of the patients with a potentially life-threatening emergency were transferred to secondary level hospitals and the mortality of those hospitalized in primary level hospitals was 3.2%, room for improvement is clearly evident. Based on our findings, improvements could be obtained by strengthening inter-hospital transfer systems, training staff in emergency/critical care skills and by making mechanical ventilation and advanced life support techniques available at the emergency rooms of primary level hospitals.

## Additional file

Additional file 1: Study Site’s Capacity to Manage Medical Emergencies. (DOCX 16 kb)
