# Supplementary material for: Management of potentially life-threatening emergencies at 74 primary level hospitals in Mongolia: results of a prospective, observational multicenter study
Source: BMC Emerg Med. 2017 May 8;17:15. doi: 10.1186/s12873-017-0127-4 (PMC5422969; doi:10.1186/s12873-017-0127-4)
Supplement: Additional file 1: — Study Site’s Capacity to Manage Medical Emergencies. (DOCX 16 kb) [file 12873_2017_127_MOESM1_ESM.docx]

**Study Site’s Capacity to Manage Medical Emergencies**

**Physician-in-charge** ____________________________________________ Date: _________________

**Number of hospital beds** ______________________ beds

**Availability of …**
… emergency or critical care physician □ yes □ no
… emergency or critical care nurse □ yes □ no
… emergency/resuscitation room □ yes □ no
… delivery room □ yes □ no
… operation theatre □ yes □ no
… high dependency/intensive care unit □ yes □ no
… sonography machine □ yes □ no
… echocardiography machine □ yes □ no
… full blood count measurement □ yes □ no
… glucometer □ yes □ no
… urine dip stick/urin analyzer □ yes □ no
… blood gas analyzer □ yes □ no

**Possibility to perform …**… wound care □ yes □ no
… burn wound care □ yes □ no
… foreign body removal □ yes □ no
… abscess drainage □ yes □ no
… fracture management □ yes □ no
… advanced cardiac life support □ yes □ no
… oxygen administration □ yes □ no
… airway management □ yes □ no
… cardioversion □ yes □ no
… thoracocentesis □ yes □ no
… paracentesis □ yes □ no
… pericardiocentesis □ yes □ no
… blood transfusion □ yes □ no
… non-invasive mechanical ventilation □ yes □ no
… invasive mechanical ventilation □ yes □ no
… central venous catheter insertion □ yes □ no
… renal replacement therapy □ yes □ no
